# Four-year-olds selectively transmit true information

**DOI:** 10.1371/journal.pone.0284694

**Published:** 2023-04-27

**Authors:** Ellyn B. Pueschel, Ashley Ibrahim, Taylor Franklin, Samantha Skinner, Henrike Moll

**Affiliations:** Department of Psychology, University of Southern California, Los Angeles, California, United States of America; Lamar University, UNITED STATES

## Abstract

Two experiments (*N* = 112) were conducted to examine preschoolers’ concern for the truth when transmitting information. A first experiment (Pilot Experiment) revealed that 4-year-olds, but not 3-year-olds, selectively transmitted information marked as true versus information marked as false. The second experiment (Main Experiment) showed that 4-year-olds selectively transmitted true information regardless of whether their audience lacked knowledge (Missing Knowledge Context) or information (Missing Information Context) about the subject matter. Children selected more true information when choosing between true versus false information (Falsity Condition) and when choosing between true information versus information the truth of which was undetermined (Bullshit Condition). The Main Experiment also revealed that 4-year-olds shared information more spontaneously, i.e., before being prompted, when it was knowledge, rather than information, the audience was seeking. The findings add to the field’s growing understanding of young children as benevolent sharers of knowledge.

## 1 Introduction

Deliberate communication of information, whether in the form of practical demonstration or propositional knowledge exchange, is a major mechanism responsible for cultural continuity [1–3]. By being shown, told, and taught things, children build their capacities for reason [4] and acquire knowledge that would otherwise be difficult or even impossible to obtain [5].

Children’s responsiveness to shared information, both in the form of pedagogy [6, 7] and testimony [8, 9], has been intensively studied. The origins of children’s own skills of sharing information, however, have only recently become a focal area of study [10] (but see [11, 12] for earlier research). Studying these origins is important because children contribute to culture’s progression not only by acquiring cultural knowledge but also by actively spreading what they know [13]. In laboratory and natural settings, preschoolers have repeatedly been shown to faithfully transmit what they have learned to siblings [14–16] and other children [17–19].

Just like children’s social learning is selective [20, 21], so is their information-sharing. From a young age, children adjust the information they transmit to the audience’s perspective [22, 23], goal [24], knowledge and expertise [25, 26], and cognitive maturity [27]. Children also factor in the information’s expected utility for the specific learner [28] and tailor their messages to the communicative setting, for example by prioritizing general information in pedagogical but not story-telling contexts [29, 30, see also 31].

This article is concerned with children’s attention to the truth in their information-sharing. Theories of cultural evolution explain the cross-generational accumulation of adaptive ideas and practices by learners’ biases toward specific models and content. For example, learners tend to copy those who are competent and whose actions are successful [32]. In the theoretical domain, success equates *knowledge* and therefore access to the truth. Testimony and teaching are communicative practices that serve to circulate knowledge [33, 34], with social learners being on the look-out for true, rather than any old, information.

Literature on the development of testimonial learning shows that children attend to truthfulness from a young age [35–37]. Even infants are epistemically vigilant, as shown by selective imitation of accurate informants [38, 39] and extended looking times when speakers mislabel objects [40]. Children’s abilities to track and identify correctness increases in the preschool years, with 3- to 4-year-olds being able to explicitly identify informants as “good” or “not good” and statements as “true” (or “right”) or “wrong” depending on whether they are truthful [37, 41, 42]. By age 4 to 5, children distrust “liars” [43] and find it unacceptable to make claims without having the necessary evidence [44].

Less is known about children’s own adherence to the truth when sharing information. Forensic psychological studies on child testimonies and cognitive-developmental research on the emergence of lying offer some insight. Studies of child witnesses in court indicate that at around age 3 or 4, children begin to be able to “take the oath” and be expected to give veridical reports [45]. Interestingly, this roughly corresponds with the age when children begin denying their moral transgressions [46, 47], suggesting that they grasp the meaning and different implications of making true versus false statements.

The courtroom and discourse about moral transgressions arguably constitute special circumstances in which agents are motivated to disclose or hide specific information. No studies we know of have investigated whether young children select true information to share when there are no biases, apart from a preference for true information, that might motivate the selection of one or another content to share.

### 1.1 The present study

In two experiments, we investigated preschoolers’ attention to the truth when transmitting information about animals to an interested audience. The experiments, which were conducted in-person and online, had two phases. In the learning phase, 3-year-olds (Pilot Experiment) and 4-year-olds (Pilot and Main Experiment) received information with different truth values (“true” vs. “wrong”, which is child-friendlier than “false” [42, 48]). In the transmission phase, children had the chance to share what they learned with a curious audience. The ages of 3 and 4 were chosen based on studies attesting that children this age can faithfully share information [49, 50] and have sufficient understanding of truth value assignments [42].

In addition to measuring whether children transmitted true or false statements, we examined their intrinsic motivation for information-sharing by identifying whether they volunteered information spontaneously, *before* the audience made a request, or non-spontaneously, in response to a request. In the Main Experiment, we further manipulated the communicative context by having the audience either avow to not “know anything” (Missing Knowledge Context) or to not “have heard anything” (Missing Information Context) about the subject. This allowed us to explore whether expressing lack of *knowledge* as opposed to (more truth-neutrally) *information* increases children’s truth-sharing. Lastly, the Main Experiment included a “Bullshit Condition” in which true information was not pitted against false information but against information the truth of which was undetermined. Bullshitting is the spreading of information with no concern for whether what is stated is true or false [49]. Adding this condition served to explore whether children grasp that adequate information-sharing requires excluding falsehoods but also information that has not been established as true.

## 2. Pilot experiment

### 2.1 Methods

#### 2.1.1 Participants

Participants were *N =* 24 (12 female) three-year-olds (*M* = 43.28 months, range = 38.3–46.9 months) and *N* = 24 (12 female) four-year-olds (*M* = 54.18 months, range = 50.3–59.2 months). Six additional children were tested but excluded due to uncooperativeness. Parents had given written consent for their child’s participation prior to the experiment, and the study was approved by the University of Southern California Institutional Review Board (UP-17-00266). Per parental report, 62% of children were White, 23% multiracial, 10% Asian and 5% African American; 51% were Latinx. Children’s socio-economic status as measured by annual household income varied from under $20,000 to above $120,000. Thirty-six children were tested in-person at a children’s museum (24) or preschool (12) and received a small toy for participation. Due to the COVID-19 pandemic, an online test format was used for the remaining 12 children, whose families received a gift card for participating.

#### 2.1.2 Materials and design

**Fig 1:**
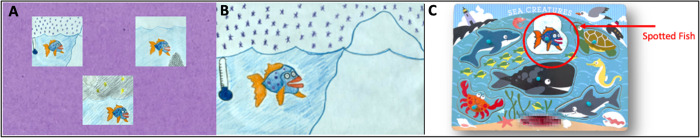
Materials from pilot experiment. Panel A: A cover page of one of the books (about the “Spotted Fish”) used in the learning phase. Cover pages showed all three pieces of information that the book contained. Panel B: One of the pages from the “Spotted Fish” book depicting the piece of information “The Spotted Fish likes cold water.” Panel C: One of the puzzles used in the transmission phase. The original animal puzzle was modified to show the fictional animal (the “Spotted Fish”). Note: A part of the image of the puzzle was intentionally blurred due to copyright restrictions. This figure serves to illustrate the object without depicting it with perfect accuracy.

A memory game served as warm-up activity for the child and the first experimenter (E1). There were three trials, each consisting of a learning and a transmission phase. In each learning phase, which the child experienced with a second experimenter (E2), two books about one fictional animal were used. Of the six books total, two were about the “Button Mouse”, two about the “Loba Lizard”, and two about the “Spotted Fish”. Each book contained three pieces of information (one piece of information per page). The books’ cover pages showed miniature images of the three pieces of information they contained, as can be seen in Fig 1. This served to help children retain the information from the learning phase in the following transmission phase (with E1), during which the books remained present. Each of three transmission phases revolved around one of three animal puzzles. One piece from each puzzle was manipulated so that it showed the fictional animal (e.g., the “Spotted Fish”) from the preceding learning phase.

In the learning phase, E2 shared with the child three pieces of “true” information (from one book) and three pieces of “wrong” information (from the other book) about a given animal. In the subsequent transmission phase, the child had the opportunity to tell E1 what they (the child) had learned. Animal order, book order, order of information within a book, and which book contained “true” vs. “wrong” information were counterbalanced.

#### 2.1.3 Procedure

E1 sat next to the child, stating that she loved animals and was curious about them. After briefly playing an unrelated memory game to build rapport, E1 said she had to leave but would return to play an animal puzzle with the child later. E1 left and E2 came in, marking the start of the learning phase.

*Learning phase*. E2 sat next to the child with both books about the same fictional animal. She placed the books on the table in random spatial order and said “Let’s learn about an animal! This is the [*animal name*, e.g., *Spotted Fish*].” E2 then picked up the first book and, depending on the counterbalancing schedule, shared information marked as true or false. For true information, E2 said “I know a few things about the [*animal name*, *e*.*g*., *Spotted Fish*], and they’re all true.” E2 flipped through the pages and, on each page, communicated a piece of information corresponding to the image on that page. Having shared all three pieces of information, E2 stated, “Those were all true.” E2 then picked up the second book and shared false information: “Someone once told me a few things about the [*animal name*, *e*.*g*., *Spotted Fish*], but they’re all wrong! Let me tell you what they said.” After sharing the false information, E2 stated “I don’t know where they heard those things, but they’re not true!” The reverse order of events occurred if children received false before true information. After completing both books, E2 placed them (with the cover-pages on top) in front of the child and exited.

*Transmission phase*. E1 returned with one of the animal puzzles. E1 placed the puzzle on the table and handed the child two puzzle pieces showing real animals. The third animal she handed to the child showed the fictional animal from the learning phase (e.g., the “Spotted Fish”). E1 asked “Oh! What’s this one called?” E1 waited for an answer. If the child did not provide the name, E1 gave the animal’s name and stated her lack of knowledge, “I don’t know anything about the [*animal name*, *e*.*g*., *Spotted Fish*].” E1 waited 10s before restating her ignorance. If after another 10s children did not share information, E1 explicitly asked for knowledge, “Do you know anything about the [*animal name*, *e*.*g*., *Spotted Fish*]?” After the child shared information, the transmission phase ended. Subsequent cycles of learning and transmission phases were as above.

For the 12 children who were tested online, the procedure was adjusted in the following way. Via Zoom, E1 expressed interest and curiosity about animals in the same way she did in the in-person procedure. After stating that she would leave and return later, E1 turned off her camera and microphone. E2 entered the screen and initiated the learning phase. The books were presented as slides and had the same layout as the physical books (cover page plus three content pages). After finishing the slides, E2 took leave. She turned her camera off and replaced her Zoom background with the books’ cover pages so that they remained visible for the child. E1 then turned her camera on and held up the puzzle board. E1 showed the puzzle pieces in turn in the same way described for in-person testing, with the third piece showing the fictional animal from the preceding learning phase. E1 used the same sequence of prompts as described above.

#### 2.1.4 Scoring and reliability

A rater scored children’s responses based on the video-recordings. For every shared piece of information, the rater judged whether it was “true” (scored “1” for correct) or “false” (scored “0” for incorrect). Children’s reproductions did not need to match original statements verbatim but only needed to preserve their meaning (e.g., “Lives near the ocean” was scored as the original information “Lives near water”). If a child said nothing or something unintelligible or not from the book, the code “other” (scored “2”) was given. For information rated as true or false (scored “0” or “1”), the rater then determined whether it was shared *before* E1’s prompt (“Do you know anything about the [*animal name*]?”) and thus spontaneous (scored “1”), or whether it was shared *after* this prompt and thus non-spontaneous (scored “0”).

To assess inter-rater-reliability, a second rater, who was ignorant of the truth or falsity of the information, scored the responses of 25% randomly selected children. First, she decided whether what children said matched information from the learning phase. If it did not match, the rater applied the score “2”. If the information matched, the rater decided whether the information was spontaneous or non-spontaneous. The rater was then given an answer key to determine whether the information was true (“1”) or false (“0”). Inter-rater reliability was excellent, both for information type shared (Kappa = .95) and for spontaneous vs. non-spontaneous information-sharing (Kappa = 1). Disagreements were resolved by discussion.

### 2.2 Results

#### 2.2.1 Information type transmitted

Generalized Linear Mixed Models (GLMMs) for repeated measures with a binomial error structure and a logit link function using the “glmer” function in the “lme4” package [52], with subject included as random effect, were run to test for effects of demographic and experimental variables. There were no effects of gender, race, socioeconomic status, information type order (true vs. false first), animal order, or trial on the type of information children shared, *p*s > .34.

**Fig 2:**
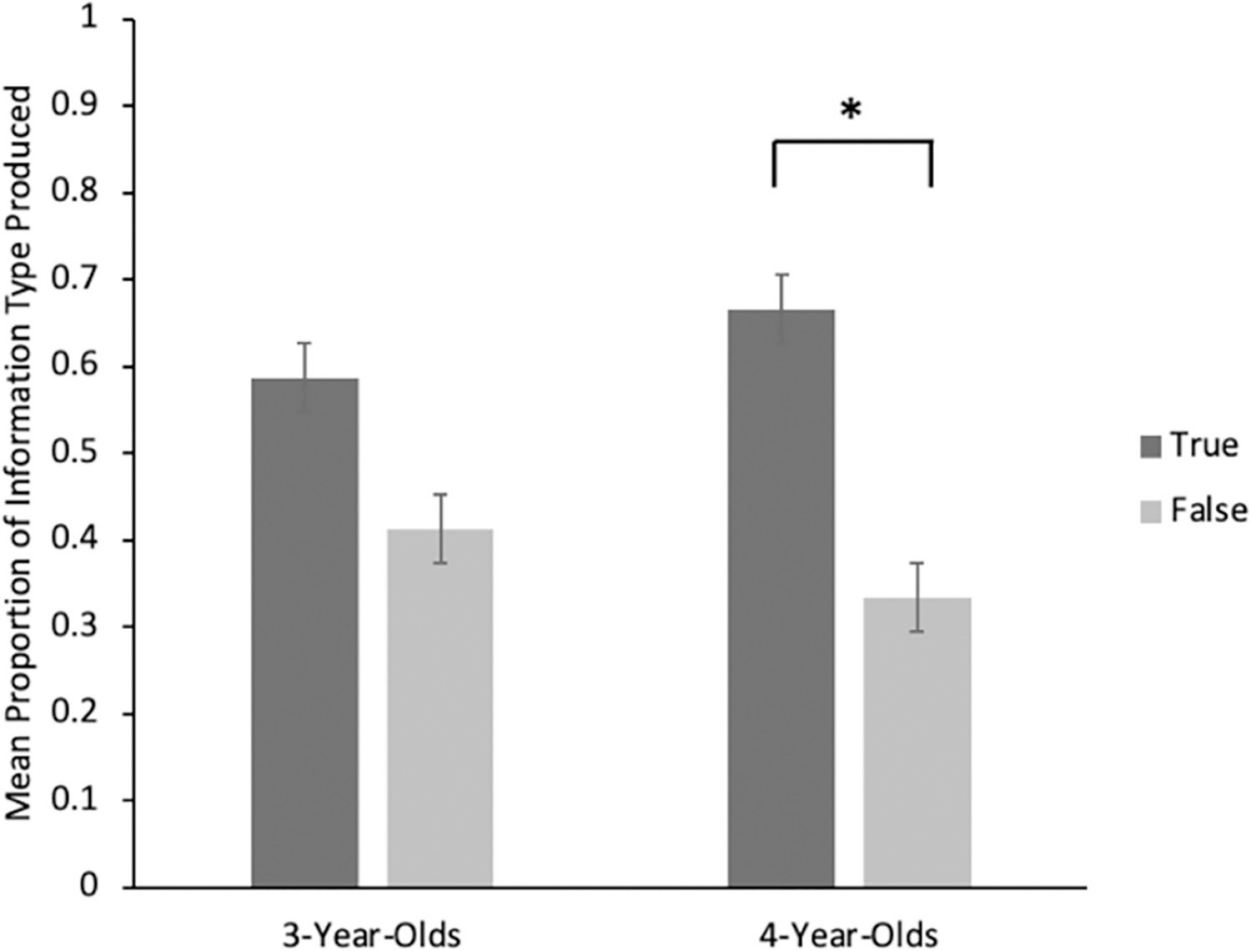
Mean proportion of information type children transmitted in pilot experiment. * p < .05.

Of the 3-year-old children, only 10 communicated any information scored as true or false (with 51 out of 72 total trials receiving a score of “2” for “other”). Due to the resulting lack of power, we only report descriptive statistics for this age group. On average, 3-year-olds transmitted 59% true and 41% false information. Of the 4-year-olds, 20 communicated information scored as either true or false (18 out of 72 trials were scored as “2”). In this age group, 68% of information children transmitted was true, and 32% was false. A paired-sample t-test showed that this was significant, *t* (19) = 2.70, *p* = < .01, *d* = .60. As seen in Fig 2, 4-year-olds transmitted more true than false information. A one-sample t-test further showed that 4-year-olds’ selection of true information significantly exceeded chance set at 50%, *t* (19) = 2.84, *p* = .01, *d* = .63.

#### 2.2.2 Spontaneity of transmission

GLMMs were run to test for effects of demographic and experimental variables (see 2.2.1). No such effects were observed, *p*s > .11.

The 3-year-olds shared 37% of information spontaneously and 63% non-spontaneously. These numbers were inverted for the 4-year-olds, who shared information spontaneously in 63% of cases and non-spontaneously in 37%. A paired-sample t-test revealed that this difference did not reach significance, *t* (19) = 1.52, *p* = .15, *d* = .34. However, a post-hoc power analysis revealed that with the given sample and effect size, a power of only .30 was detected. The results are therefore inconclusive.

### 2.3 Discussion

In this pilot experiment, we measured whether 3- and 4-year-olds preferably transmit information introduced as true over information introduced as false. The task proved too difficult for 3-year-olds, since only 40% shared any information. Rather than revealing that 3-year-olds do not understand the connection of communication and truth, the fact that over half of the children did not respond indicates that the research paradigm is not suited for children this young. Similarly high rates of non-responding in 3-year-olds were observed by Baer and Friedman [25] in their information-sharing task. Alternative measures, such as action demonstrations or the exchange of very simple propositions are advised for this age group. By age 4, children are not only skilled at transmitting propositional information, as evidenced by high response rates, but they also show a clear concern for the truth. Furthermore, 4-year-olds often shared information spontaneously—before the audience asked for it—suggesting that they are intrinsically motivated to transmit what they know.

Although this pilot project offers tentative insights into children’s attention to the truth when spreading information, what can be gleaned from it is limited by several factors. The most severe limitation comes from the fact that not all children in the final sample, especially the 3-year-olds, shared information. Second, the pandemic-related transition from in-person to online testing near the end of the experiment led to a lack of procedural sameness. Finally, the experiment leaves open unaddressed questions about the conditions under which children commit themselves to the truth. What if the audience, rather than presenting herself as lacking *knowledge*—which, by definition, implies truth—claims in more truth-neutral fashion not to have heard anything about the subject matter? Would varying the communicative context affect children’s truth bias? Another question pertains to “bullshitting” [51] defined as the sharing of information regardless of its truth. While this experiment indicated that 4-year-olds steer clear of false information, it is unknown whether they also avoid “bullshitting” by only asserting what has been established as true. These issues were addressed in the next experiment, which was conducted exclusively online.

## 3. Main experiment

In this online experiment, 4-year-olds (*N* = 64) were placed in either of two communicative contexts. In one context, the audience stated that she lacked knowledge (Missing Knowledge) and in another, she declared that she had not heard anything (Missing Information) about the subject. To investigate the robustness of children’s adherence to the truth, we compared a condition in which true was pitted against false information (Falsity Condition) with a condition in which true information was contrasted with information with no definite truth value (“Bullshit” Condition). As Frankfurt [51] showed, bullshitting is spreading information without regard for its truth or falsity. Adding this condition allowed us to test whether preschoolers grasp that cooperative information-sharing precludes not only the sharing of falsehoods but also of information that may or may not be true.

### 3.1 Methods

#### 3.1.1 Participants

A power analysis revealed that with a power of .80 and alpha of .05, a sample of *N* = 34 in each communicative context was required to detect a medium effect (d = .5). For design-related reasons, we decided on *N* = 64, with 32 (16 female) children in the Missing Knowledge Context (*M* = 54.42 months, range = 50.10–59.5 months) and 32 (16 female) in the Missing Information Context (*M* = 52.43, months, range = 49.6–58.3 months). Eleven more children were tested but excluded due to uncooperativeness. Parents had given written consent prior to the experiment, and the study was approved by the University of Southern California Institutional Review Board (UP-17-00266). Per parental report, 63% of the children were White, 1% African American, 16% Asian, 13% multiracial, and 7% “other”; 17% of all children were Latinx. Children’s socio-economic status, measured by annual household income, varied from below $20,000 to above $120,000. Parents received a gift card for participation.

#### 3.1.2 Materials and design

As in the Pilot Experiment, two books about one animal (learning phase) and a matching puzzle (transmission phase) were used in each trial. A fourth animal, the “Sparie Bird”, was added and thus a fourth trial was added. The names “Spotted Fish” and “Button Mouse” were changed to “Merkle Fish” and “Dango Mouse” to use entirely fictional names. Children were randomly assigned to either the Missing Knowledge Context or the Missing Information Context. All children received two trials of the Falsity Condition and two trials of the Bullshit Condition. In addition to the factors mentioned in the Pilot Experiment, condition order was counterbalanced.

#### 3.1.3 Procedure

*Learning phase*. The experiment began with E1 stating that she loved and was curious about animals. E1 then exited (turned off her camera) and E2 entered with two books. When sharing a book with true information, E2 stated “I know a few things about the [*animal name*, *e*.*g*., *Sparie Bird*]. They’re all true. Here’s what I know.” After sharing all three pieces of information, she stated, “Those things are all true.” When sharing a book with false information (Falsity Condition), E2 said “I heard a few things about the [*animal name*, *e*.*g*., *Sparie Bird*]. They’re all wrong. Here’s what I heard.” After sharing all three pieces of false information she said “Those things are all wrong.” When sharing a book with information of undetermined truth value (Bullshit Condition), E2 said “I heard a few things about the [*animal name*, *e*.*g*., *Sparie Bird*]. I don’t know if they’re true or wrong. Here’s what I heard.” Having shared all pieces of information, she said “Maybe those things are true, maybe those things are wrong”.

*Transmission phase*. As in the Pilot Experiment, E1 returned with a puzzle and held up the pieces one by one. When holding up the third piece with the fictional animal, E1 stated “I don’t know anything about [*animal name*, *e*.*g*., *Sparie Bird*]” in the *Missing Knowledge Context*, and “I didn’t hear anything about the [*animal name*, *e*.*g*, *Sparie Bird*]” in the *Missing Information Context*. If a child did not respond within 10s, E1 repeated her statement. If the child still did not volunteer information for the next 10s, E1 explicitly requested knowledge or information by asking “Do you know anything about the [*animal name*, *e*.*g*, *Sparie Bird*]?” (Missing Knowledge Context) or “Did you hear anything about the [*animal name*, *e*.*g*, *Sparie Bird*]?” (Missing Information Context). After the child responded or was silent for 10s, E1 took leave, thereby terminating the transmission phase. Subsequent cycles of learning and transmission phases were as described.

#### 3.1.4 Scoring and reliability

The scoring and reliability procedures were the same as in the Pilot Experiment. For the Bullshit Condition, information with no determined truth value was coded as incorrect (scored “0”) and true information as correct (scored “1”). Inter-rater reliability was excellent, both for information type transmitted (Kappa = .97) and for the spontaneity of transmission (Kappa = .97). Disagreements were resolved by discussion.

### 3.2 Results

In 196 out of 256 total trials, children produced answers that were coded as true or false (Falsity Condition) or as true or “truth undetermined” (Bullshit Condition). The remaining trials were scored “2” for “other” (25 of these trials were from the Missing Knowledge Context and 35 trials were from the Missing Information Context). The following analyses are based on the 196 valid trials.

#### 3.2.1 Information type transmitted

GLMMs were run to test for effects of the same demographic and experimental variables as in the Pilot Experiment. There was an effect of information type order, *p* < .03, with children transmitting more true information when they had received this information prior to the alternative information (false or truth-undetermined) in the learning phase. No other effects were detected, *p*s > .13.

**Fig 3:**
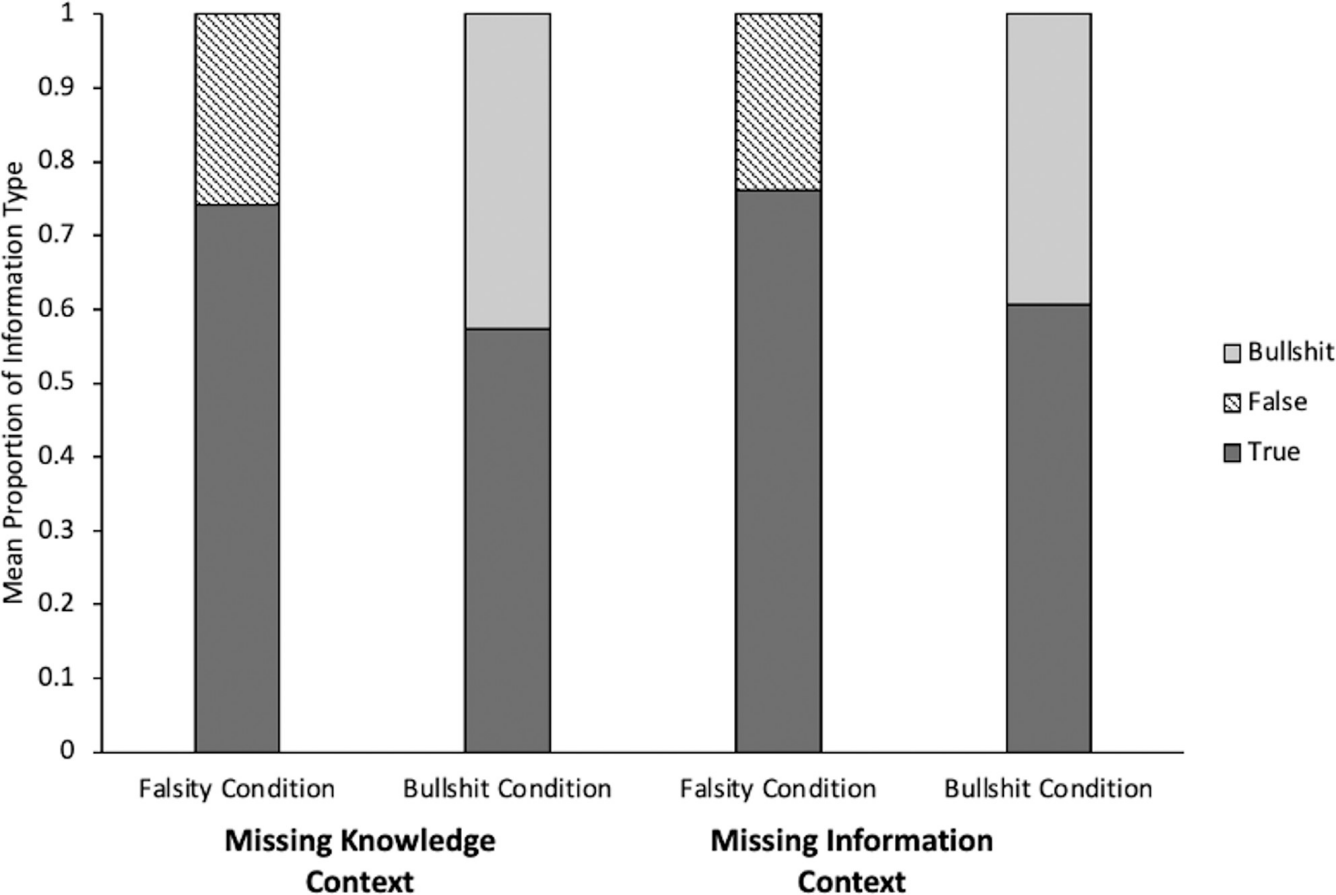
Mean proportion of information type transmitted in main experiment broken down by communicative context and condition.

A GLMM with communicative context (Missing Knowledge vs. Missing Information) and condition (Falsity vs. Bullshit) as predictors and transmitted information type as a dependent variable was run to test whether condition or communicative context influenced the rate of true information shared. There was no effect of communicative context, *p =* .58, and no interaction, *p* = .52, but there was a condition effect, β = -.46, *p* < .01, with children sharing more true information in the Falsity than the Bullshit Condition. One sample t-tests (with Bonferroni-adjusted p-values) showed that despite the difference between conditions, children chose true statements above chance both, when selecting true against false information, *t* (56) = 7.34, *p* < .001, *d* = .97, and when selecting true information against information of undetermined truth value, *t* (53), 2.75, *p* = .026, *d* = .35. Fig 3 shows the mean proportion of information type transmitted in each context and condition.

#### 3.2.2 Spontaneity of transmission

GLMMs were run to test for the same demographic and experimental variables. There was a significant gender effect, with boys responding more spontaneously than girls, β = 2.20, *p* < .02, and a significant trial effect, with children answering more spontaneously as trials progressed, β = .54, *p* < .001. No other effects were observed, *p*s > .58.

**Fig 4:**
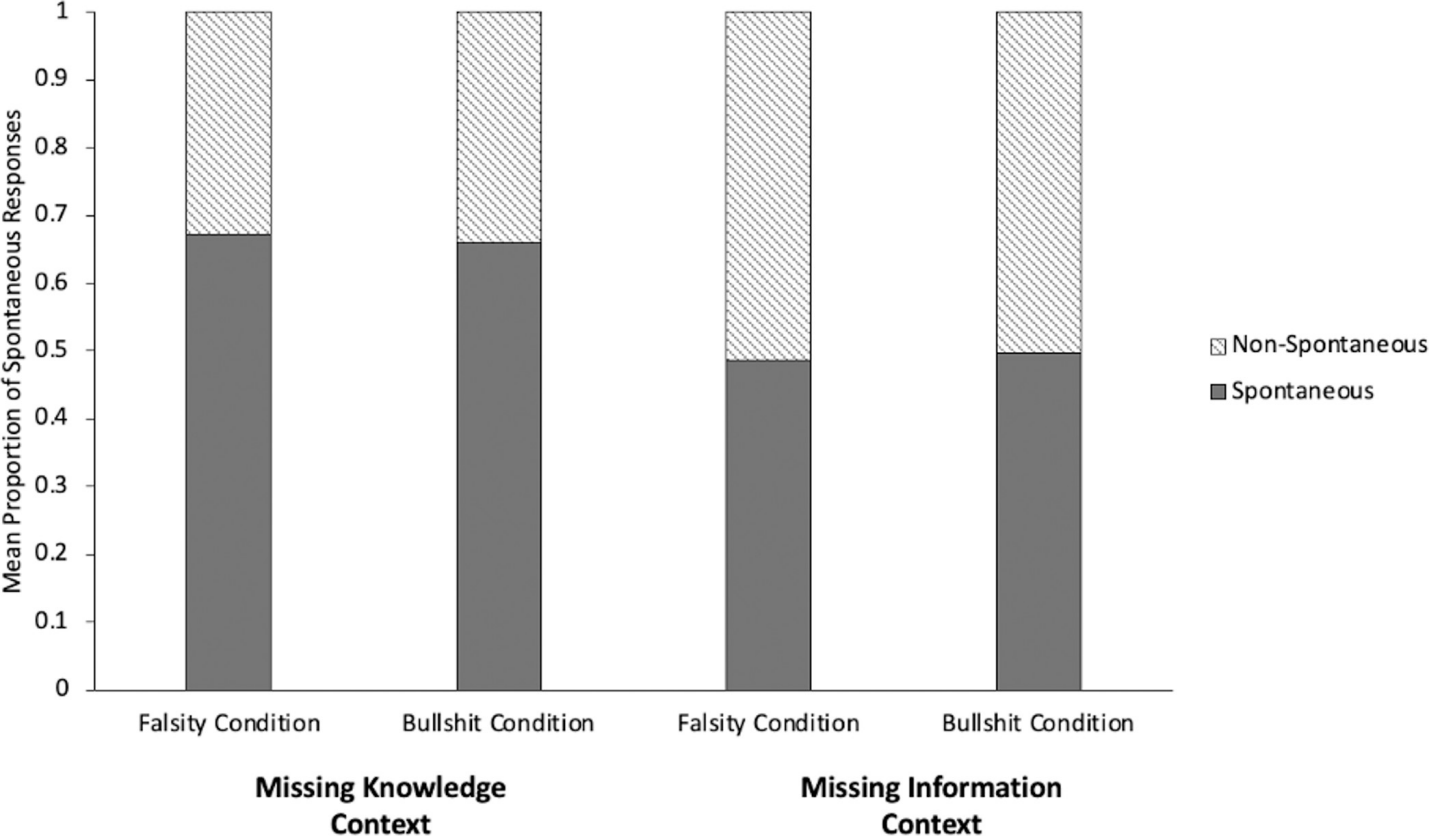
Mean proportion of spontaneous information transmitted in main experiment broken down by communicative context and condition.

A GLMM with context (Missing Knowledge vs. Missing Information) and condition (Falsity vs. Bullshit) as predictors of spontaneity was conducted. The model showed no condition effect, *p* = .17, and no interaction between context and condition, *p* = .20. There was, however, a significant effect of context, β = 2.61, *p* = .04, with children sharing information more spontaneously when the audience lacked knowledge than when she lacked information. Fig 4 shows the proportion of spontaneous information transmitted in each context and condition.

## 3.3 Discussion

This experiment confirmed the Pilot Experiment’s observation that 4-year-olds selectively transmit true information. Children’s preference for the truth pervaded contexts in which the audience purported to lack *knowledge*—and, thus, by definition, true information—and those in which the audience lacked information of any kind. The audience’s use of the factive “know” to express her ignorance was thus not responsible for children’s truth bias, as children were equally committed to the truth when the audience (more neutrally) stated that she had not heard anything about the subject. What *did* differ between epistemic and informational contexts, however, was the spontaneity with which children relayed information. Children volunteered information more promptly when the audience expressed a lack of knowledge than when she stated she had no information. What might explain this context effect? The separately observed gender difference, whereby boys shared information more spontaneously than girls, cannot account for the context effect because gender was balanced across contexts. We assume that for young children, satiating another’s thirst for knowledge is more urgent than is filling an information gap because having knowledge is of intrinsic value, whereas the value of information depends on whether it is useful or relevant.

Although 4-year-olds transmitted true statements both, when selecting true against false information and when selecting true information against information of unknown truth value, they adhered to the truth more strongly when the other information was outright false. This reflects either of two possibilities. One is that 4-year-olds have greater tolerance for spreading information the truth of which is questionable than they have for spreading outright falsehoods. In other words, 4-year-olds might find it permissible to circulate information that might turn out to be true. The other possibility is that children did not fully grasp the ambiguity of information introduced as “maybe true or maybe wrong”. Perhaps some children failed to understand the uncertainty and instead took the information to be either true or false, with the effect that overall, more children chose this information than information clearly brandmarked as “false”.

## 4. General discussion

This study investigated young children’s attention to the truth when sharing information. In the Pilot Experiment, 4-year-olds selectively shared true as opposed to false information and tended to share information spontaneously, before their audience requested it. For many 3-year-olds, the task of restating propositions proved too difficult, indicating that an information-sharing paradigm of the kind used here is not suited for children this young (the same was found by Baer and Friedman [25]). Future investigations of knowledge transmission in toddlers should thus turn to alternative methods. One possibility is to measure children’s demonstrations of practical knowledge, as is done in diffusion chain studies [see 53]. Alternatively, one might let children gesture toward physical representations of information, such as information presented in a book.

Our study contributes to the field’s growing recognition that young children are not only avid social learners [54] but also remarkable givers of knowledge and information in their own right. Experimental studies confirm what naturalistic observations [e.g., 16, 55] have long suggested, which is that not only adults and youth but also young children contribute to the propagation of knowledge in the community [13]. Even toddlers select specific information to share. Vredenburgh et al. [56] showed that 2-year-olds are more likely to demonstrate to others what they themselves learned in a pedagogical setting than what they learned outside of pedagogy. By age 4 to 5, children flexibly inform and instruct others by tailoring their message to the audience and context. For example, preschoolers adjust the quantity, complexity, and generality of information to the learner’s background knowledge and maturity level [25] or to the type of conversation they are having [29, 30]. Preschoolers are not just sensitive to the learner’s knowledge but also to their own. This is suggested by a study by Kim et al. [57], who found that 3- and 4-year-olds refrained from giving information and instead showed gestures of uncertainty when they lacked sufficient knowledge to communicate. A recent study furthermore suggests that by around age 7, children adequately evaluate teachers or informants based on the quantity and quality of information they share with a learner [58].

The specific contribution the present study makes is to show that children stay away from false information and even, albeit to a lesser extent, from information that has not been established as true. That young children filter out (potential) falsehoods and selectively spread veridical information shows their understanding that listeners want facts, not “noise”. By selecting true information to share, children follow Grice’s [59] cooperative principle of communication. In particular, they satisfy the maxim of quality, whereby speakers should only assert what they take to be true or have evidence for.

Research on testimonial learning has confirmed that although tending to trust others, children are epistemically vigilant from an early age, preferring informants with a track record of being reliable and accurate [20, 37, 60]. Our study shows that children express the same vigilance when they are on the giving end of the exchange. When relaying information, children weed out falsehoods and carefully select information that is veridical. Children thus seek knowledge, rather than mere opinion, and they express their concern for the truth both as learners and as sharers of information.

One might assume that preschoolers’ selective information-sharing piggybacks on their theory of mind skills, which also develop at around age 4 [61, 62]. Preschoolers’ newly acquired theory of mind skills would, in this account, allow them to consider what the listener needs or wishes to learn. Support for this idea comes from correlational studies showing that preschoolers’ teaching skills are associated with their understanding of other minds [49, 63, 64]. However, tying selective information-sharing to a theory of mind might be problematic because 3-year-olds and younger children are underrepresented in selective information-sharing tasks, most likely due to their already discussed difficulties with the task requirements. Finding simpler transmission paradigms is thus key not only for pinpointing the onset of selective-information sharing but also for improving our understanding of the relation between selective information-sharing and a theory of mind.

We conclude with a bigger-picture reflection on the nexus between knowledge and communication. In their skillful navigation of the world, humans rely on knowledge, rather than instinct, as do most other animals. Unlike instinct, knowledge is not innately given but acquired. And the way in which knowledge is acquired, as epistemologists and learning theorists have pointed out [65, 66], is not by solitary discovery but by the communicative practices of teaching and testimony. Without aspiring to the truth and seeking to enlarge the pool of shared knowledge, human communication would miss its aim and fail to meet its evolutionary purpose, which is to improve one another’s orientation in and understanding of the world. The present study adds to evidence that even young children have a hunch that communication is a vehicle of knowledge.
